# Mate Preference of Female Blue Tits Varies with Experimental Photoperiod

**DOI:** 10.1371/journal.pone.0092527

**Published:** 2014-03-26

**Authors:** Laura B. Reparaz, Kees van Oers, Marc Naguib, Claire Doutrelant, Marcel E. Visser, Samuel P. Caro

**Affiliations:** 1 Department of Animal Ecology, Netherlands Institute of Ecology (NIOO-KNAW), Wageningen, The Netherlands; 2 Behavioural Ecology Group, Department of Animal Sciences, Wageningen University (WUR), The Netherlands; 3 Department of Evolutionary Ecology, CEFE-CNRS, Montpellier, France; Pennsylvania State University, United States of America

## Abstract

Organisms use environmental cues to time their life-cycles and among these cues, photoperiod is the main trigger of reproductive behaviours such as territory defence or song activity. Whether photoperiod is also important for another behaviour closely associated with reproduction, mate choice, is unknown. In many bird species, mate choice occurs at two different times during the annual cycle that strongly differ in daylength: in late winter when photoperiod is short and social mates are chosen, and again around egg-laying when photoperiod is longer and extra-pair mates are chosen. This duality makes the role that photoperiod plays on mate choice behaviours intriguing. We investigated the effect of photoperiod on mate choice using three experimental photoperiodic treatments (9 L:15 D, 14 L:10 D, 18 L:6 D), using blue tits (*Cyanistes caeruleus*) as a biological model. We show that female choice was stronger under long photoperiods. In addition, female blue tits spent significantly more time near males with long tarsi and long wings. This latter preference was only expressed under long photoperiods, suggesting that some indices of male quality only become significant to females when they are strongly photostimulated, and therefore that females could select their social and extra-pair mates based on different phenotypic traits. These results shed light on the roles that photoperiod may play in stimulating pair-bonding and in refining female selectivity for male traits.

## Introduction

In temperate zones, most animal species start breeding remarkably regularly each year. The short time-window dedicated to reproduction generally occurs in spring, when temperatures are mild and large amounts of nutrient- and protein-rich food necessary for raising offspring become available. To time and organize the complex temporal pattern of reproductive behaviours, animals use environmental cues that predict, well in advance, the onset of suitable conditions for breeding [1], [2]. Among these cues, the most reliable predictive signal is the annual change in photoperiod: every year, favourable spring conditions for breeding coincide with a progressive increase in day length. Exposure to increasingly longer photoperiods triggers a cascade of neuroendocrine reactions that prepare animals for breeding: e.g., genes are activated in the brain, reproductive hormones increase in concentration in the blood, and gonads mature and start producing gametes [3]–[6]. These physiological changes in turn stimulate the expression of a complex and well-organized suite of reproductive behaviours such as territory defence, courting, nest preparation and communication displays, like songs in songbirds [1].

Another behaviour that is closely associated with reproduction is mate choice. Mate choice is a key life-history decision that impacts an individual's current reproductive success and fitness [7]. Both direct and indirect benefits, such as parental care, genetic quality for the offspring or access to a high quality territory, can be derived from selecting an appropriate partner [8]. As mate choice is a crucial prerequisite for breeding, one might predict that it is influenced by environmental factors and mechanisms similar to those that determine reproduction, including photoperiod [9]. Long photoperiods could enhance an animal's selectivity for particular traits that are only relevant in a sexual context, and not outside the breeding cycle [10], [11]. In meadow voles (*Microtus pennsylvanicus*) for instance, exposing females to long days is sufficient to reverse their winter-typical preference for female odours to a summer-typical preference for male odours [12]. In birds, female canaries (*Serinus canaria*) are responsive to male song only after being exposed to long photoperiods, an effect that intesifies when they reach fertility [13].

In many bird species, including those considered monogamous, individuals mate with more than one partner within a single breeding season. In the blue tit (*Cyanistes caeruleus*), the subject of the current study, up to 68% of females engage in copulations with males that are different from their social partner [14], [15]. The choice to engage in extra-pair copulations is made around egg-laying [16]–[19], while the social mate is typically chosen much earlier, in late winter [20], [21]. This illustrates that mate choosing behaviours are not only important before reproduction starts, but also during the breeding season, for selecting individuals with whom to potentially engage in extra-pair copulations. Given that these two time periods strongly differ in their day length, the role that photoperiod plays on mate choice behaviours in these species is intriguing. The potential photoperiodic modulation of female selectivity for male traits could lead to differences in the criteria used by females for choosing social mates in late winter, and extra-pair mates during longer days in the breeding season. In great tits (*Parus major*), for instance, females select extra-pair mates during the breeding season based on personality traits [22], while there is no clear evidence that they do so for their social mates that are chosen under a shorter photoperiod [23], [24].

Here, we examine the relationship between photoperiod and mate choice behaviours in captive blue tits. The birds we study originate from the long-term Corsican study sites of Muro and Pirio, which strongly differ in their habitat characteristics and selection pressures [25]. Birds from these two sites fundamentally vary in a number of characteristics, including laying dates that occur one month apart [26]–[28]. Experiments in captivity have suggested that these population differences in reproductive traits resulted from a different response to photoperiod [29], [30]: early breeding birds from Muro have a lower response threshold to photoperiod (i.e., respond to shorter photoperiods) than late-breeding birds from Pirio. By specifically investigating the effect of photoperiod on mate choice between Muro and Pirio birds, population-specific patterns can be identified that shed light on photoperiodic effects on mate choice in general. In particular, if Muro birds have a lower response threshold to photoperiod, we expect mate choice-related behaviours to be enhanced by comparatively shorter photoperiods in Muro birds than in Pirio birds.

The primary aim of this study therefore is to identify the impact of photoperiod and origin population on the general interest of females in males and on their strength of preference for a particular male. Because females attend to various male traits, and because a female's choice could be influenced by her own traits [31], we quantified female mate choice in relation to both male and female biometric traits (tarsus and wing lengths) that reflect their intrinsic qualities [16]. Moreover, we explore how personality [22], [32] influences both male attractiveness and female decision rules, and whether these are dependent on the photoperiod to which birds are exposed. If birds choose different kinds of mates (social *vs.* extra-pair) based on different indices of quality, we predict that some of these morphological and behavioural traits will only affect mate choice behaviours under some photoperiods and not others.

## Methods

### Ethics Statement

The experiments run in this study were approved by the Animal Experimentation Committee of the Royal Dutch Academy of Sciences (DEC-KNAW; permit number CTE.09–04 and NIOO11.09). The work performed in the field was approved by the prefectural office of Corsica and the Regional Direction of Environment (DIREN) committee (permit numbers 2009–0379 and 3467).

### Subjects

We used sixty-seven second calendar year (hatched in 2010) captive Corsican blue tits (34 females, 33 males) for the experiment, all originating from Muro or Pirio populations in Corsica. These populations are located at similar latitudes, and therefore experience similar absolute, and annual variation in, photoperiods. Whole broods of chicks (N = 7 broods at Muro, 10 broods at Pirio) were collected from their nests when they were 10-day-olds, and were transferred to the laboratory for standardized hand rearing [33], [34]. Briefly, birds were transported to the Netherlands Institute of Ecology (NIOO-KNAW) by car in natural nests in three wooden boxes (30×14 cm and 10 cm high divided into three compartments, each containing one nest). These three boxes were placed into a larger wooden box that helped maintain temperature and humidity at optimal levels for chicks of this age (around 25°C and 50% humidity). If necessary, electric warming pads (ThermoLux, Murrhardt, Germany) were placed under the large box to provide extra heating. During the travel, and later on at the institute, chicks were fed every half-hour, for 14 hours per day (7:00 am–9:00 pm), with a diet consisting of a mixture of curd cheese, ground beef heart, baby cereal, multivitamin solution and calcium carbonate, supplemented with wax moth larvae and bee larvae, until independence. After reaching independence (about 35 days after hatching), the birds were transferred to an individual home cage of 0.9×0.4 m×0.5 m high with solid bottom, top, side and rear walls, a wire-mesh front and three perches.

As part of a previous experiment, half of the birds had been gradually experiencing a lengthening photoperiod (14 L:10 D), by increments of 38 minutes per week, while the other half of the birds had been moved directly to a constant long photoperiod (14 L:10 D). At the beginning of the present experiment, therefore, birds were photostimulated with a 14 L:10 D light schedule. These pre-treatments did not impact any of the response variables measured in the present experiment (all P-values >0.05), and will not be further considered.

For the present experiment, all birds were housed individually in cages (identical to those described above) in single-sex rooms. Temperature in the rooms was kept between 15 and 17°C. Birds had visual and auditory contact with each other. Diet consisted of a mixture of egg, cow heart, dry bird food, vitamins, and minerals, supplemented with dry food containing insects, peanuts, and vitamins. Birds also had access to mealworms, grit, and sunflower seeds. Food and water were provided *ad libitum*.

At the end of the experiment, birds that were not used for other research purposes were released into their population of origin in Corsica. This reintroduction took place in mid-March 2012, a time when temperatures are mild in Corsica and food is abundant. During transportation, adult birds were housed in wooden boxes (100×30 cm×15 cm high, divided into 6 compartments, one bird per compartment) where they received their normal diet (see above).

### Novel Environment Test

Directly before the start of the experiment, birds were tested using the novel environment test modified from Verbeek et al. [35], an established operational measure of avian personality. All individuals were tested individually in a room (4.0×2.4×2.5 m) with five artificial wooden ‘trees’. Test-subjects were housed adjacent to the test room, in cages equipped with sliding doors leading to the test room. Test-subjects were placed in the cages 30–120 minutes before testing. The test-subject was introduced into the room without being handled, by darkening the subject's cage with a draped towel, and opening the sliding door that connected the cage to the test room. After the sliding door was opened, the observer turned on the light in the test room. Once the test-subject entered the room, its movements were observed for two minutes, after which the trial ended, and the subject was returned to its home cage. The total number of movements between the five trees was counted, as well as hops up, down, and along branches of each tree, and based on these measures, birds were given an exploratory score on a continuous scale with higher scores indicating faster exploration, and lower scores indicating slower exploration. Exploration scores of birds in our experiment varied from 10 (very slow) to 92 (very fast).

### Photoperiodic treatments and pairing

Blue tits from both Muro and Pirio were exposed to three different photoperiodic treatments (9, 14, and 18 hours of light per day - 9 L:15 D, 14 L:10 D, and 18 L:6 D). These photoperiods respectively correspond to the minimum photoperiod encountered in winter in Corsica, the photoperiod at which Corsican blue tits breed, and an artificially long photoperiod often used in captive experiments investigating the effect of day length on birds' behaviour and physiology [36], [37], [38]. Photoperiodic treatments were applied sequentially to all birds, as space limitations prevented a set-up in which the three different treatments were run simultaneously to different groups of birds housed in different rooms. As a consequence, the effect of photoperiod was confounded with date (but see below).

At the start of the experiment, birds were exposed to a 14 L:10 D schedule, for two weeks (see above). During the last two days of the 14 L:10 D schedule, a group of 11 females (consisting of both Muro and Pirio birds) was mate choice tested. After this first group of females had been tested, the photoperiod of the single-sex rooms was switched to 18 L:6 D, and at the end of two weeks, another group of 11 (previously-untested) females was tested over two days. The photoperiod of the single-sex rooms was then switched to the final treatment, 9 L:15 D. At the end of two weeks at 9 L:15 D, a final group of 12 (previously-untested) females was mate choice tested. Thus, a total of 34 females were mate choice tested in this experiment, meaning that the females that were tested under one given treatment were never re-used later. See table S1 for a summary of female mate choice testing by origin population and photoperiodic treatment. Two weeks of treatment is sufficient to elicit stable and robust neuroendocrine responses and their associated behaviours in birds. In Japanese quails (*Coturnix coturnix japonica*), for example, brain activity and circulating luteinizing hormone (LH) concentrations start increasing within hours after the transfer to long photoperiods and reach their maximum within a few days [39]. In starlings (*Sturnus vulgaris*), maximal concentrations of luteinizing and follicle stimulating hormones (FSH) are reached after only one week of exposure to long photoperiods, and plasma vitellogenin (a major egg-yolk precursor produced by the liver in response to gonadal steroids [40]) has been shown to significantly increase within two weeks of photostimulation [41]. Finally, blue tits can lay eggs only three weeks after having been transferred to long photoperiods [29]. Thus, an interval of two weeks between the changes in photoperiod and the behavioral tests appears to be sufficient for birds to be in a relatively stable physiological state at the time of testing.

Each female was exposed to a pair of unrelated, previously un-encountered males for mate choice-testing. Twelve pairs of males were chosen (six from Muro, and six from Pirio) for the experiment. Each pair was formed with two males from the same origin population. In the majority of the pairs, a male with a high-exploratory score was paired with a male with a low-exploratory score. However, some pairs were closely matched in terms of exploratory scores due to a limited number of males with differing scores being available for pairing. All females were matched with a pair of males from their own origin population, and all females were tested with males undergoing the same photoperiodic treatment. We deliberately chose to expose both males and females to the same photoperiods as, in the wild, birds of both sexes are always simultaneously exposed to identical photoperiods. Testing female choice for males that are in a completely different physiological state than that of the female would thus have been highly artificial. We quantified male activity and behaviours while in the presence of a female to assess the effect of photoperiod on male behaviours. No effects were found (see table S2), and therefore the effect of photoperiod on female behaviours is not driven through its effect on male behaviours. While each female was only tested once, due to a limited number of available males, each male pair was used in two to four mate choice trials.

The different photoperiods followed each other during the course of the experiment. This means that the effect of daylength was confounded with the effect of date. Females' choice might therefore have been affected by this date effect, especially if it was their endogenous clock, rather than photoperiod, that was triggering the observed behavioural changes. Note however, that the photoperiods experienced by the birds mimicked the natural order of photoperiodic changes for a bird in spring (i.e., long - very long - short photoperiod). Thus, like in the real world, the birds were exposed to different daylengths at different times of the year, and therefore any influence of the endogenous clock that would occur here, would also occur in the wild. In addition, we tested a different group of females in each of the photoperiodic treatments (i.e., individual females were tested only once), meaning that there is no order effect due to females tested multiple times in the same succession of treatments. Finally, birds remained at least two weeks under each photoperiod before behavioural testing occurred, which is sufficient to ellicit robust and relatively stable changes in physiological states, with a minimal impact of past photoperiods (see above).

### Mate choice testing

A mate choice test chamber was designed for this study, based primarily on previous designs by Kurvers et al. [42] and Naguib et al. [43]. Two identical test chambers were built in two separate rooms. Each test chamber consisted of a large neutral area (2.4×2.5 m) equipped with three wooden artificial trees, two identical male zones (a semi-enclosed area in front of each male cage where the female could choose to be, and in which she was only able to see that male) (0.95×0.95 m), each equipped with one wooden tree, and a small area between the two male cages with a door for the tester to pass through (Fig. S1). Each male cage (0.85×0.40×1.0 m) was constructed of wood and wire mesh, and affixed to a rolling base. A ‘natural sun’ light bulb (Arcadia Compact Bird Lamp 20 W, Arcadia Products, Redhill, United Kingdom) was installed in the top of each male cage to allow for male UV-coloration visibility, and each male cage was equipped with four wooden perches. A curtain was installed between the male cages and the male zones to conceal the males from the female as needed throughout the testing (Fig. S1). Three closed-circuit spy cameras (Henelec DF-117, London, United Kingdom) were affixed in each test chamber, with one in the neutral area, and one in each of the two male zones, to capture female activity throughout the trial. Footage from the cameras was recorded in a digital format using a hand-held video recorder wired to the cameras (Archos 604, Igny, France).

Between 10 and 120 minutes before the start of the mate choice trial, the female test-subject was transferred from the home cage to a temporary cage adjacent to the test chamber. The procedure to house the birds and transfer them between the cages and the test rooms was the same as for the scoring of personality (see above). Males were brought into the test chamber male cages by the tester, and were not visible to the female, remaining concealed behind curtains. The birds were then given approximately 10 minutes to acclimate to the test chamber. After the acclimation period, the curtains were raised, and the first half of the mate choice trial took place for 20 minutes. After that time, the curtains were closed, and the male cages were switched, in order to account for any female side-bias. The second half of the trial took place over 20 minutes. Thirty-four females were each tested in one of the two mate choice chambers. After testing, subjects were returned to their home cages. Each female was tested only once, except in two cases in which there was equipment failure, and those females were re-tested at the end of the experiment with a pair of previously un-encountered males.

### Data analysis

Mate choice was inferred from the amount of time a female spent near a particular male. Spatial proximity and duration are reliable indicators of choice, and have been shown to correlate with female responsiveness and aviary pairing in other species [44]. Other commonly studied behaviours in mate choice contexts, such as singing activity or copulation sollicitation displays, are very rarely expressed by blue tits in captivity (pers. obs.), and thus were not quantified here. Video footage from the mate choice tests was analyzed using the computer program Observer XT (version 10.5, Noldus, Wageningen, The Netherlands). Footage was analyzed in terms of time females spent in each of the three possible zones (neutral, right male, left male). Data from the two trials were pooled together for statistical analyses.

#### Female interest and female preference strength

The primary estimations of mate preference calculated were ‘interest’ and ‘preference strength’. In the calculations that follow, each male from the mate choice tests was defined as “male 1” or “male 2”. These labels were arbitrary: male 1 was always the male whose first location in the mate choice test was the right-hand cage of the test chamber. As the variables described below are proportions, they were arcsine square root transformed to achieve normality.

*Female interest* was indicative of a female's overall motivation to spend time near either male in the pair of males she was exposed to (and was not indicative of a particular choice for one male), and was calculated as follows:
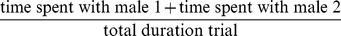



where the “total duration trial” includes both the time spent with each of the two males, and the time the female spent in the neutral zone, away from any male (see Fig. S1).

*Female preference strength* indicated the strength of a female's choice for one male out of the two males she encountered in the test. Female preference strength is defined as the relative amount of time spent with the chosen male (the male with which the female spent >50% of the male-zone time) compared to the time spent with both males, and calculated as follows:
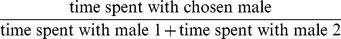



Statistical analyses regarding preference strength were weighted by female interest. In other words, females who spent more time with males in general were given more statistical weight in the analyses than females who were less interested in males.

#### Effects of photoperiod and population of origin on female interest and female preference strength

To examine how photoperiod and population of origin could explain variation in female interest in males, and preference strength, two linear mixed-effect models were used (one for interest and one for preference strength). In each model, photoperiodic treatment (treated as a continuous variable), origin population, their interaction, and time of day birds were tested, were used as fixed factors. Test room and male pair were treated as random factors. The models were simplified using stepwise backward elimination of the non-significant terms, starting with higher-order interactions. P-values were obtained by model comparisons between a model that included the term of interest, and a model that excluded this term.

#### Effect of male and female traits on preference strength

To examine how specific behaviours (exploration score) and morphological traits (tarsus and wing lengths) affect preference strength, and how these traits might interact with photoperiod in modulating the preference, we constructed different linear mixed-effect models, each with the analogous factor for the female, the chosen male, and the non-chosen male. It was important to examine the characteristics of the chosen male and the non-chosen male simultaneously because both determined where the female spent her time. It was also important to take the characteristics of the females into account as these could modulate their choice, especially if assortative mating occurs [31]. Three linear mixed-effect models were made, each with preference strength as the dependent variable, and photoperiodic treatment as a main effect, together with a combination of effects that depend on the behaviour or morphological trait of interest (tarsus length, wing length, and exploration score; see table S6 for details). Male pair and test room were fitted as random variables. The procedures for model simplification and for obtaining P-values were the same as described above.

#### Statistical software

Statistics were carried out using the lmer (package lme4) procedure in R version 2.14.1 (R Development Core Team 2011). All tests were two-tailed and an alpha of 0.05 was applied throughout.

## Results

### Effects of photoperiod and population of origin on female interest

On average, females spent more than half of their time in the male areas (see table S3 for descriptive data). Photoperiodic treatment and population of origin had no significant effect on female interest for males (all *P*-values >0.15, table S4). After stepwise backwards elimination of non-significant terms, none of the factors included in the analysis significantly explain the time females spent close to either male, relative to the time they spent in the neutral zone.

### Effects of photoperiod and population of origin on female preference strength

**Figure 1:**
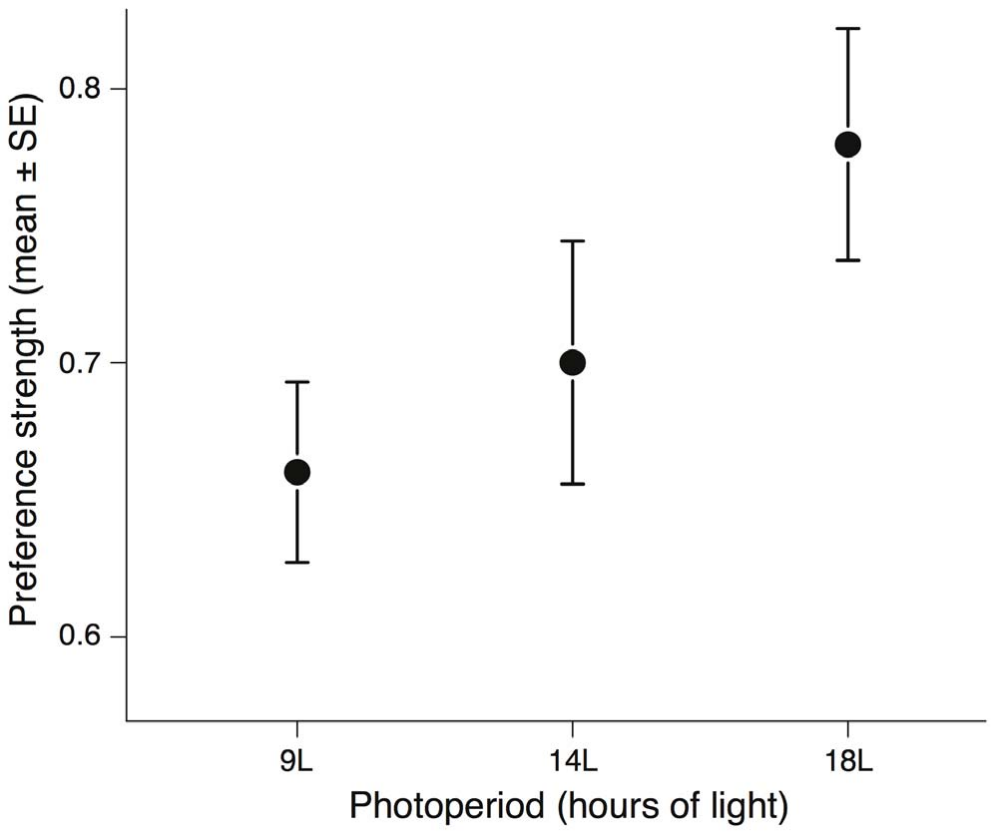
Effect of photoperiodic treatment on mean preference strength. Female preference strength increased as photoperiodic treatment increased (n = 34, p = 0.018). Preference strength indicates the strength of a female's choice for one male out of the two males she encountered in the test.

Photoperiodic treatment significantly affected female preference strength (*t* = 2.254, *P* = 0.018; table S5). The longer the photoperiod, the stronger the female preference became (Fig. 1). Blue tits from Muro and Pirio, however, did not differ in their preference strength (*t* = 1.296, *P* = 0.155) and this preference was similarly affected by photoperiod in both populations (*t* = 0.659, *P* = 0.449; table S5).

### Effect of male and female traits on female preference strength

**Figure 2:**
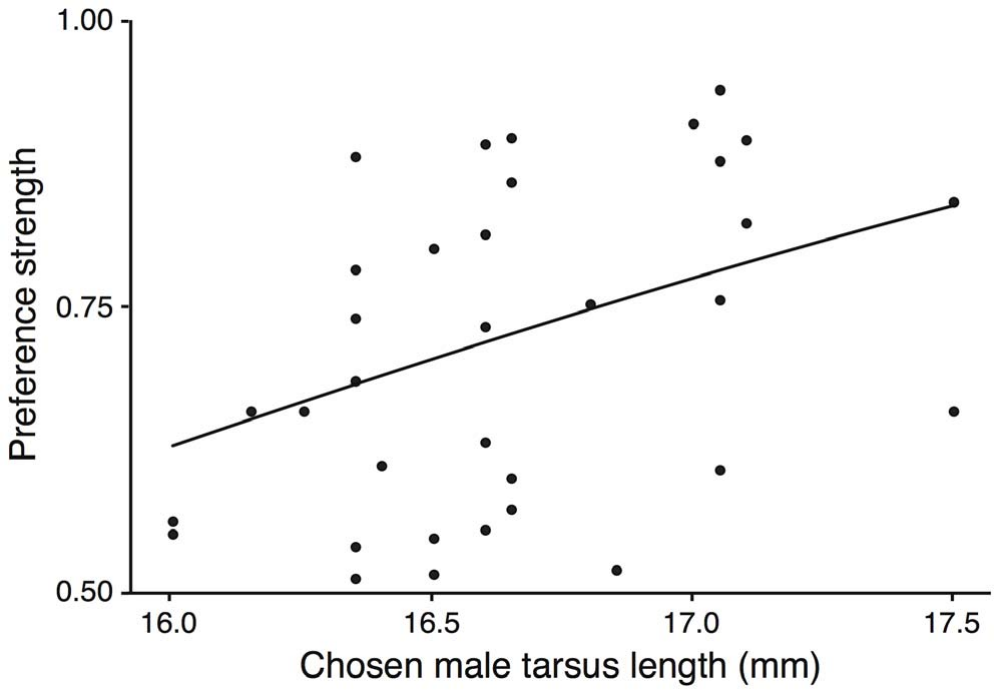
Effect of chosen male tarsus size on female preference strength. Females spent significantly more time (stronger preference) with males with longer tarsi (n = 34, p = 0.012). Each point on the graph represents one tested female. Tarsus length is a measure of skeletal size in birds and is often considered as an indicator of overall quality.

**Figure 3:**
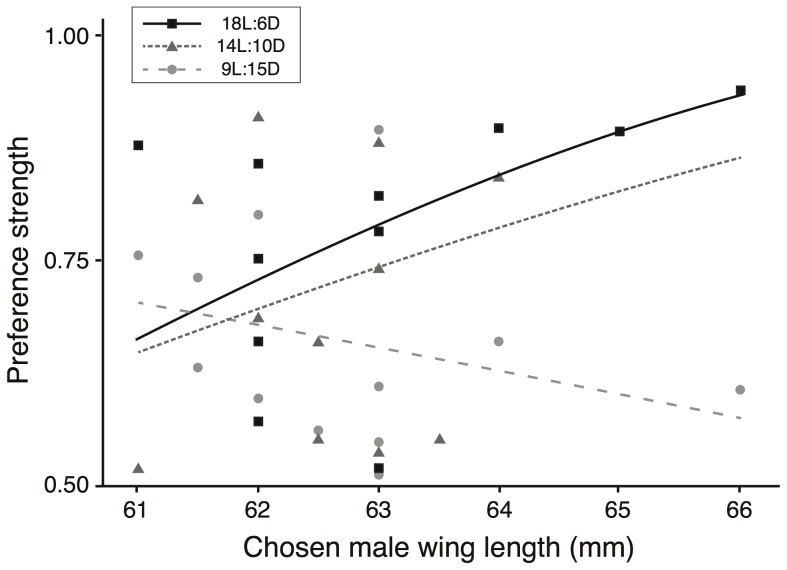
Interaction between chosen male wing length and photoperiod on female preference strength. Females spent more time with males with longer wings as photoperiod increased (n = 34, p = 0.015). Wing length varies between moults, and is often considered as an indicator of seasonal or annual quality in males.

Female Corsican blue tits spent significantly more time close to the male they had chosen if that male had long tarsi (*t* = 2.376, *P* = 0.012; see Fig. 2 and table S6). Similarly, females spent more time with the male they had chosen if that male had long wings; but this preference was dependent on the photoperiodic treatment, as shown by the significant interaction between photoperiodic treatment and chosen male wing on female preference strength (*t* = 2.162, *P* = 0.015; see Fig. 3 and table S6). In this same analysis, the wing length of the non-chosen males and of the females did influence the female preference strength (table S6). Personalities of both the males and females did not siginficantly influence the strength of female choice (table S6).

## Discussion

This study experimentally investigated how photoperiod affects mate choice in blue tits. We show that preference strength (proportion of time spent with the male chosen) was significantly influenced by photoperiod, with females exhibiting stronger preferences when photoperiod was longer. In addition, males with long tarsi and wings were more attractive to females than smaller males. Particularly interesting is the result that the strength of a female preference for long male wings was dependent on photoperiod, suggesting that photoperiod modulates female neural and behavioural selectivity, and male attractiveness (see below).

For most animals living in temperate and arctic zones, the increasing photoperiod in late winter and spring correlates reliably with forthcoming improvement of environmental conditions to a level that supports the energetic and nutritional requirements for successful reproduction [1], [3]. Photoperiod thus acts as a cue signalling the approach of suitable conditions for breeding, and many species use that cue to orchestrate their reproductive behaviours. Here, our data suggest that mate choice-related behaviours are also influenced by photoperiod, with females strengthening the choice for one particular male when photoperiod is longer. How photoperiod is causally linked to mate choice behaviours remains unclear, but our results suggest that photoperiod modulates selectivity for some male traits as indicated by the significant interaction between photoperiod and male wing length (see table S6 and Fig. 3). While males with long tarsi are attractive whatever the photoperiod, the attractive character of long wings is only apparent when photoperiod is long. This suggests that in the wild, blue tits might adopt different mate choice strategies at different times of the year, depending on daylength. In blue tits, short winter photoperiods are associated with the time social partners are chosen [20], [21], while longer spring photoperiods are associated with the time many females seek extra-pair mates [17], [19]. According to our results, social mate choice would be affected by tarsus length, while extra-pair mate choice would be affected by both tarsus and wing lengths.

Tarsus length is a reliable measure of skeletal size in birds and is typically used as an indicator of overall genetic and/or developmental quality (often in concert with body mass) [45]–[47]. Our results confirm the results of Kempenaers et al. [16] that female blue tits preferentially mate with males with longer tarsi. Tarsus length has a heritable component, and by choosing males with longer tarsi, females may produce offspring with longer tarsi [48], [49]. Individuals with longer tarsi may be better able to compete for resources due to overall larger size [16]. Interestingly, earlier studies involving the two blue tit populations used here, have shown that (i) males from Muro, which are significantly heavier and larger than males from Pirio, dominate Pirio males in captivity, and that (ii) song repertoire size is positively correlated with tarsus length in both populations [50]. In the context of our results, this could imply that females prefer males that are more socially dominant, and likely able to procure more resources, or, if tarsus indeed reflects an overall higher quality, offer better genes for offspring. Tarsus length is fixed at 15 days of age [48], whereas wing length varies between moults, and is more indicative of changes in the bird's age, health and quality on a seasonal/annual level [46], [51], [52]. Wing length could be a good indicator of parasitic infection and disease resistance, with males with longer wings being more resistant to parasites, and therefore, of better quality [51]–[54].

How photoperiod would modulate female selectivity and/or male attractiveness in blue tits is unknown, but several studies in humans, songbirds and frogs have demonstrated that high levels of steroid hormones in females facilitate mate choice-related behaviours [9], [55]–[58]. These hormones are part of the neuroendocrine cascade that an increasing photoperiod triggers in spring and could therefore mediate the modulatory effects of photoperiod on mate choice observed here. High levels of oestrogens would render females more decisive in their choices and sensitive to particular male traits, like wing length in the present case.

An additional variable investigated in this experiment was personality. The personality of a female was not found to significantly affect her interest or her preference strength. Moreover, the personality of the males to which the females were exposed had no significant effect on female choice. These results are surprising, considering that past studies have suggested that personality does play a role in mate choice [22], [23]. Those conclusions, however, arose from correlative studies conducted in the wild (but see [59]), and it is likely that in a natural setting, more factors play a role in mate formation than mate choice alone.

No reliable population differences were found between Muro and Pirio birds in interest or preference strength. This finding contrasts with our predictions and is somewhat surprising, as the Muro and Pirio populations have evolved different phenotypic and behavioural properties, such as timing of breeding, clutch size, behavioural dominance, and song features [26], [60], [61] in response to the spatial variation in their habitats. Mate choice has been shown to vary according to current or past environmental conditions [31], [62] (but see [63], [64]); we could therefore have expected similar variation in mate choice patterns between populations originating from contrasted environments [65]. On the other hand, in captive Muro and Pirio birds, the differences in some of these traits were found to disappear during long-day photoperiodic treatment [66]. In addition, recent experiments investigating the role of photoperiod on gonadal development failed to show any difference between the populations (S.P. Caro and M.E. Visser, unpub. data). As a consequence, the physiological mechanisms that orchestrate pre-breeding development might be controlled in the same way by photoperiod in the two populations, and because mate preference behaviours are photoperiod-dependent, the motivation for choosing a mate might be similarly controlled in both populations.

The sequential nature of the treatments to which the birds were exposed prevent us from fully excluding any date effect that could have been confounded with daylength. Several birds in the last treatment (9 L:15 D) could, for example, have been photorefractory at the time of testing, or some females might have been stressed by the succession of photoperiods they experienced, which in turn would have influenced their behaviours. However, we assess any such effect to be small. First, keeping female songbirds under short photoperiods, whatever their photoperiodic state (photosensitive or photorefractory), results in low oestrogen levels [67], [68], which are suspected to play a significant role in the transduction of the photoperiodic effects oberved on the behaviour of the females (see above). In particular, low estradiol levels are expected to result in a diminution of the cognitive abilities, and by extension, to lead to a lower choice discrimination [9], [55]–[58], which is what we observe here. Second, among the different traits recorded in this study, only preference strength did vary with treatment and in the predicted direction, while female interest, male and female levels of behavioural activity did not, which suggests that birds were not stressed or in unnatural physiological states, and by extension, that photoperiod *per se* is an important factor in mate choice. Finally, and as stated earlier, birds in the wild also experience different photoperiods at different dates. Females choosing social and extra-pair mates in nature, also assess them at different periods of the year.

Besides highlighting the role of photoperiod in mate choice decisions, our study also suggests that males of higher quality, as indicated by tarsus length, are generally more attractive, and that long photoperiods reveal stronger preferences for males with long wings. This modulation of mate preference indicates that different mate choice criteria could be used at different times of the year, and by extension that social and extra-pair mates could be chosen based on different phenotypic traits. This idea expands on the current literature on the mechanisms underlying the evolution on muliple traits in mate choice [69] and more studies are now needed to confirm this conclusion and intergrate more traits like song and colour.

## Supporting Information

Figure S1 **Schematic representation of mate-preference test chamber.**
(PDF)

Table S1 **Sample sizes.**
(PDF)

Table S2 **Effect of photoperiod on male behavior.**
(PDF)

Table S3 **Female descriptive data (interest, preference strength and activity).**
(PDF)

Table S4 **Effects of photoperiod and population of origin on female interest.**
(PDF)

Table S5 **Effects of photoperiod and population of origin on female preference strength.**
(PDF)

Table S6 **Effects of photoperiod, morphological and behavioural traits on female preference strength.**
(PDF)
