# Supplementary material for: Mate Preference of Female Blue Tits Varies with Experimental Photoperiod
Source: PLoS One. 2014 Mar 26;9(3):e92527. doi: 10.1371/journal.pone.0092527 (PMC3966787; doi:10.1371/journal.pone.0092527)
Supplement: Figure S1 — Schematic representation of mate-preference test chamber. (PDF) [file pone.0092527.s001.pdf]

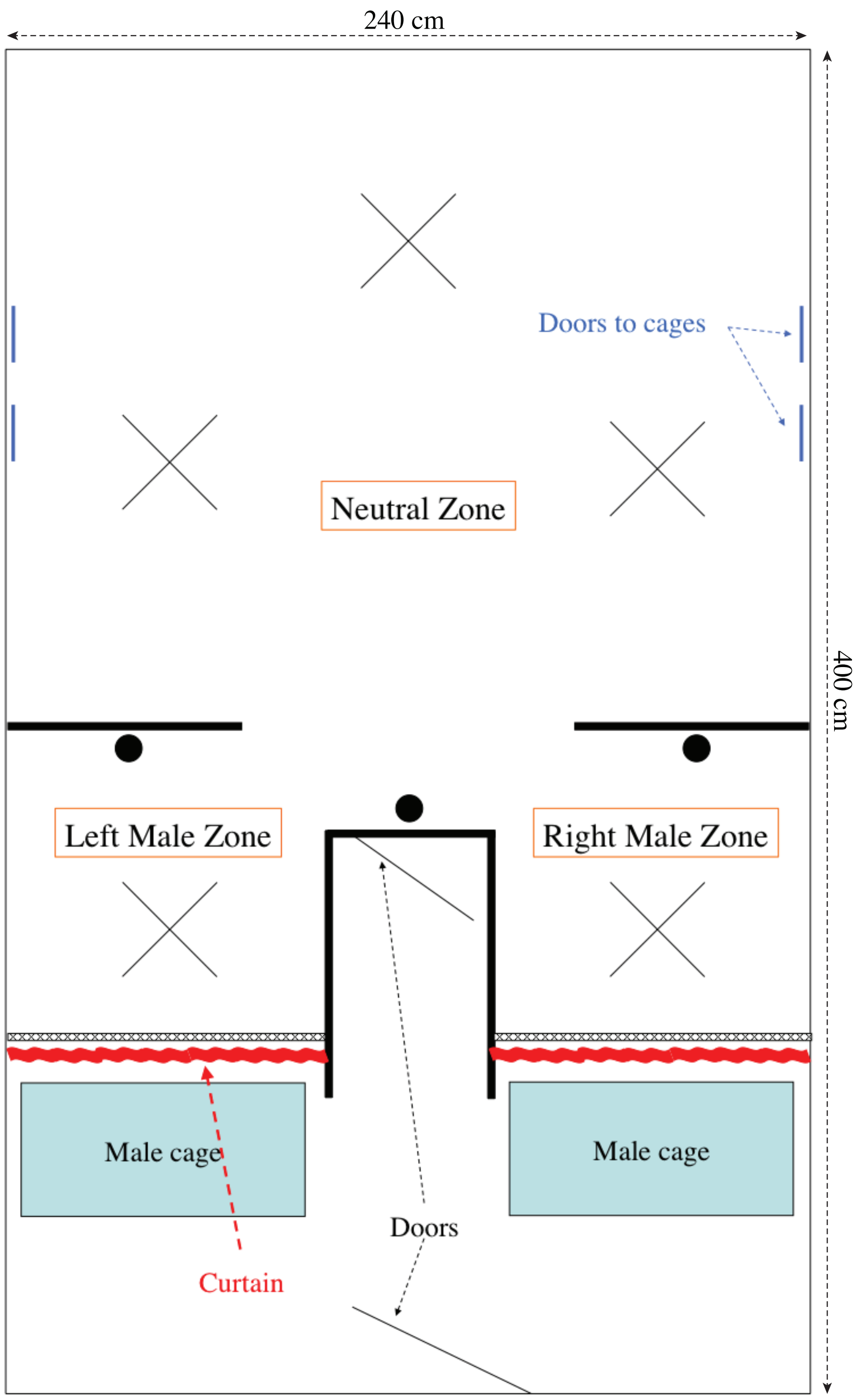

Figure S1. Mate-preference test chamber. Blue rectangles represent male cages on wheels, equipped with UV light. A curtain (red line) conceals the males from the female before the start of testing. Filled black circles represent cameras. Crosses represent perches. Males are unable to see one another through the solid sides of the cages, but the front and back of the cages are wire mesh. The female can enter the semi-boxed “Left Male Zone” or “Right Male Zone” to visit a male. Females can also choose to remain in the “Neutral Zone”, where they will not interact with a male.
