# Supplementary material for: Mate Preference of Female Blue Tits Varies with Experimental Photoperiod
Source: PLoS One. 2014 Mar 26;9(3):e92527. doi: 10.1371/journal.pone.0092527 (PMC3966787; doi:10.1371/journal.pone.0092527)
Supplement: Table S1 — Sample sizes. (PDF) [file pone.0092527.s002.pdf]

Table S1. Sample sizes used in the mate-choice experiment in Corsican blue tits, indicating number of females tested within origin population, and photoperiodic treatments under which testing occurred

Photoperiodic treatment	Muro Females	Pirio Females	Totals
9L:15D	6	6	12
14L:10D	4	7	11
18L:6D	5	6	11
**Totals**	**15**	**19**	**34**
