# Supplementary material for: Mate Preference of Female Blue Tits Varies with Experimental Photoperiod
Source: PLoS One. 2014 Mar 26;9(3):e92527. doi: 10.1371/journal.pone.0092527 (PMC3966787; doi:10.1371/journal.pone.0092527)
Supplement: Table S2 — Effect of photoperiod on male behavior. (PDF) [file pone.0092527.s003.pdf]

Table S2. Effect of photoperiod on male behavior in Corsican blue tits (n=24). We used mixed-models ("lme", package nlme in R), with photoperiod as a fixed-factor and male identity as a random intercept, to test whether males behave differently under different photoperiods. In the first model, the response variable is the general activity of the males (average number of body movement per second). In the second model, the response variable is the occurrence of a common sexual display in tits (average number of short wing-shivering bouts per second, see [1]). In both cases, the minimal adequate model is the null model, thus photoperiod did not affect male behaviours.

Trait analyzed	Variable	Estimate	df	t	P
General activity					
	Photoperiod	-0.002	1	-0.269	0.795
Wing-shivering					
	Photoperiod	0.0006	1	0.858	0.386

Reference

1. Hinde RA (1952) The behaviour of the great tit (*Parus major*) and some other related species. Behaviour Supplement: III-201.
