# Supplementary material for: Mate Preference of Female Blue Tits Varies with Experimental Photoperiod
Source: PLoS One. 2014 Mar 26;9(3):e92527. doi: 10.1371/journal.pone.0092527 (PMC3966787; doi:10.1371/journal.pone.0092527)
Supplement: Table S3 — Female descriptive data (interest, preference strength and activity). (PDF) [file pone.0092527.s004.pdf]

**Table S3.** Female data showing averages of interest, preference strength, and female activity near males for the three photoperiodic treatments (9, 14 and 18 hours of light). Female activity near males is a count variable that includes female movement within the male area including any kind of hopping and flying, and excluding exiting the male area for the neutral area. Data for analyses were arcsine square root transformed to achieve normality; the data presented in this table were calculated from raw, untransformed data.

| Trait analyzed | 9L:15D | 14L:10D | 18L:6D |
|---|---|---|---|
| Interest (mean ± SD) | 0.54±0.24 | 0.52±0.24 | 0.68±0.21 |
| Preference strength (mean ± SD) | 0.32±0.23 | 0.40±0.29 | 0.56±0.28 |
| Activity near males (mean number of body movements ± SD) | 114±48 | 91±50 | 85±51 |
