# Supplementary material for: Mate Preference of Female Blue Tits Varies with Experimental Photoperiod
Source: PLoS One. 2014 Mar 26;9(3):e92527. doi: 10.1371/journal.pone.0092527 (PMC3966787; doi:10.1371/journal.pone.0092527)
Supplement: Table S4 — Effects of photoperiod and population of origin on female interest. (PDF) [file pone.0092527.s005.pdf]

Table S4. Effects of photoperiod and population of origin on female interest for males (= proportion of time spent with any male) in Corsican blue tits (n=34). The minimal adequate model is the null model, variables are presented in the reverse order in which they were removed from the model.

Variable	Estimate	df	t	P
Photoperiod	0.016	1	1.323	0.180
Origin	0.135	1	1.429	0.143
Time of day	-0.001	1	-0.019	0.850
Photoperiod*Origin	0.017	1	0.689	0.433
