# Supplementary material for: Mate Preference of Female Blue Tits Varies with Experimental Photoperiod
Source: PLoS One. 2014 Mar 26;9(3):e92527. doi: 10.1371/journal.pone.0092527 (PMC3966787; doi:10.1371/journal.pone.0092527)
Supplement: Table S5 — Effects of photoperiod and population of origin on female preference strength. (PDF) [file pone.0092527.s006.pdf]

Table S5. Effects of photoperiod and population of origin on female preference strength (= proportion of time spent with the male chosen) in Corsican blue tits (n=34). Photoperiod is the only variable in the minimal adequate model, eliminated variables are presented in the reverse order in which they were removed from the model.

Variable	Estimate	df	t	P
**Photoperiod**	**0.016**	**1**	**2.254**	**0.018***
Origin	0.070	1	1.296	0.155
Time of day	-0.001	1	-0.378	0.669
Photoperiod*Origin	0.009	1	0.659	0.449
