# Supplementary material for: Mate Preference of Female Blue Tits Varies with Experimental Photoperiod
Source: PLoS One. 2014 Mar 26;9(3):e92527. doi: 10.1371/journal.pone.0092527 (PMC3966787; doi:10.1371/journal.pone.0092527)
Supplement: Table S6 — Effects of photoperiod, morphological and behavioural traits on female preference strength. (PDF) [file pone.0092527.s007.pdf]

Table S6. Effects of photoperiod, morphological and behavioural traits of males and females on female preference strength (= proportion of time spent with the male chosen) in Corsican blue tits (n=34). Variables in bold represent the minimal adequate models, eliminated variable are presented in the reverse order in which they were removed from the model.

Trait analyzed	Variable	Estimate	df	t	P
Tarsus length					
	**Photoperiod**	**0.017**	**1**	**2.495**	**0.008***
	**Chosen male tarsus**	**0.162**	**1**	**2.376**	**0.012***
	Female tarsus	-0.057	1	-0.840	0.344
	Non-chosen male tarsus	0.018	1	0.236	0.786
	Chosen male tarsus*Non-chosen male tarsus	-0.285	1	-1.223	0.158
	Photoperiod*Chosen male tarsus	0.015	1	0.767	0.363
	Photoperiod*Non-chosen male tarsus	-0.013	1	-0.630	0.445
	Photoperiod*Chosen male tarsus*Non-chosen male tarsus	-0.093	1	-1.378	0.094
	Female tarsus*Chosen male tarsus*Non-chosen male tarsus	-0.013	1	-0.891	0.263
Wing length					
	**Photoperiod**	**0.013**	**1**	**1.965**	**0.025***
	**Female wing**	**0.029**	**1**	**1.737**	**0.048***
	**Chosen male wing**	**0.034**	**1**	**1.558**	**0.079**
	**Non-chosen male wing**	**-0.044**	**1**	**-1.859**	**0.035***
	**Photoperiod*Chosen male wing**	**0.011**	**1**	**2.162**	**0.015***
	Chosen male wing*Non-chosen male wing	-0.006	1	-0.207	0.804
	Photoperiod*Non-chosen male wing	-0.001	1	-0.054	0.948
	Female wing*Chosen male wing*Non-chosen male wing	-0.001	1	-0.626	0.439
	Photoperiod*Chosen male wing*Non-chosen male wing	0.003	1	0.539	0.496
Personality					
	**Photoperiod**	**0.016**	**1**	**2.254**	**0.018***
	Female personality	-0.001	1	-0.556	0.537
	Chosen male personality	-0.001	1	-0.159	0.857
	Non-chosen male personality	-0.001	1	-0.560	0.520
	Photoperiod*Chosen male personality	0.001	1	1.385	0.111
	Chosen male personality*Non-chosen male personality	0.001	1	0.145	0.770
	Photoperiod*Non-chosen male personality	-0.001	1	-0.345	0.675
	Female personality*Chosen male personality*Non-chosen male personality	0.001	1	1.871	0.080
	Photoperiod*Chosen male personality*Non-chosen male personality	0.001	1	0.473	0.515
